# A Smart Home Energy Management System Using Two-Stage Non-Intrusive Appliance Load Monitoring over Fog-Cloud Analytics Based on Tridium’s Niagara Framework for Residential Demand-Side Management

**DOI:** 10.3390/s21082883

**Published:** 2021-04-20

**Authors:** Yung-Yao Chen, Ming-Hung Chen, Che-Ming Chang, Fu-Sheng Chang, Yu-Hsiu Lin

**Affiliations:** 1 Department of Electronic and Computer Engineering, National Taiwan University of Science and Technology, Taipei 106335, Taiwan; yungyaochen@gapps.ntust.edu.tw; 2 Department of Electrical Engineering, Ming Chi University of Technology, New Taipei City 243303, Taiwan; mhchen@mail.mcut.edu.tw; 3 Business Development Department, First International Computer, Inc. (FIC), Taipei 11491, Taiwan; jeremy_chang@fic.com.tw; 4 Customer Support & RMA Team, First International Computer, Inc. (FIC), Taipei 11491, Taiwan; sean_chang@fic.com.tw; 5 Graduate Institute of Automation Technology, National Taipei University of Technology, Taipei 106344, Taiwan

**Keywords:** artificial intelligence, cloud computing, demand-side management, edge computing, energy management system, non-intrusive appliance load monitoring, smart houses

## Abstract

Electricity is a vital resource for various human activities, supporting customers’ lifestyles in today’s modern technologically driven society. Effective demand-side management (DSM) can alleviate ever-increasing electricity demands that arise from customers in downstream sectors of a smart grid. Compared with the traditional means of energy management systems, non-intrusive appliance load monitoring (NIALM) monitors relevant electrical appliances in a non-intrusive manner. Fog (edge) computing addresses the need to capture, process and analyze data generated and gathered by Internet of Things (IoT) end devices, and is an advanced IoT paradigm for applications in which resources, such as computing capability, of a central data center acted as cloud computing are placed at the edge of the network. The literature leaves NIALM developed over fog-cloud computing and conducted as part of a home energy management system (HEMS). In this study, a Smart HEMS prototype based on Tridium’s Niagara Framework^®^ has been established over fog (edge)-cloud computing, where NIALM as an IoT application in energy management has also been investigated in the framework. The SHEMS prototype established over fog-cloud computing in this study utilizes an artificial neural network-based NIALM approach to non-intrusively monitor relevant electrical appliances without an intrusive deployment of plug-load power meters (smart plugs), where a two-stage NIALM approach is completed. The core entity of the SHEMS prototype is based on a compact, cognitive, embedded IoT controller that connects IoT end devices, such as sensors and meters, and serves as a gateway in a smart house/smart building for residential DSM. As demonstrated and reported in this study, the established SHEMS prototype using the investigated two-stage NIALM approach is feasible and usable.

## 1. Introduction

Recent distinct disciplines and breakthrough technologies, such as the Internet of Things (IoT), artificial intelligence (AI), big data analytics, and fog (edge)-cloud computing, are trending upwards in today’s modern technologically driven society. These technologies are increasingly becoming fundamental constituents of cities, such as in smart houses, buildings, and factories. Electricity is a vital resource that supports day-to-day activities and lifestyles, powering important infrastructure and services conducted for and used by customers [1]. Worldwide energy consumption is expected to significantly increase over the next few years [1,2]. Unlike alternative energy sources, such as wind and solar energy, which are clean and renewable, fossil fuel exploitation to meet ever-increasing electricity demands has a strong environmental impact on global warming and climate change. In the last few years, traditional power grids have been reinforced into intelligent, highly reliable, and fully automated grids [3]. The “smart grid” paradigm copes with ever-increasing electricity demands by customers from downstream sectors of a grid, i.e., residential, commercial, and industrial sectors, to name but a few. In a smart grid, smart meter data analytics holds tremendous potential for power utilities to (1) understand the power demands of their customers and (2) manage, plan, and optimize their power grid to be efficient and smart. Smart buildings, which integrate ongoing technological IoT advancements with AI in a smart city in a smart grid, represent a research branch of ubiquitous computing that involves the incorporation of AIoT (AI across IoT) technologies into buildings to improve customers’ comfort, healthcare, safety, security, and energy efficiency [1,4,5]. AIoT technologies support the intrusive deployment of controllers, sensors, actuators, and other advanced metering devices that make smart buildings possible and realizable. Among different types of buildings, smart houses have received great research interest, particularly from the point of view of energy efficiency [1].

In smart houses, the advent of AIoT has enabled the development of smart home energy management systems (HEMSs), relying on multiple IoT end devices such as sensors/meters and actuators networked in the Internet. These devices communicate via the Internet with a cloud-centered server serving as cloud computing, which is used for data science analytics to optimize electricity demands based on demand response (DR) prices. Most of the existing solutions in [6,7,8,9,10,11,12,13,14,15,16,17,18] have featured the viewpoints of HEMSs. However, the authors of [6,7,8,9,11,13,18] implemented the HEMSs based on cloud computing. The drawbacks of such a HEMS implemented by a centralized paradigm of AIoT will be pointed out later in this section. Taking advantage of advanced metering infrastructure (AMI), which allows bi-directional interaction between both demand and supply sides of residential customers and power utilities for grid information retrieved from and gathered in smart grids, power utilities can manage grid resources and infrastructure at a household level in an optimal way. This kind of load management is particularly valuable for power utilities, as it allows power utilities to predict future electricity demands and address ever-increasing electricity demands requested by residential customers. Smart houses using a HEMS that optimizes residential energy consumption in response to DR signals to target efficient load management are one of the key enablers in a smart grid, not only by supporting home energy management in terms of grid resources and infrastructure, but also by contributing to energy efficiency overall. Conducted in a smart house, which is equipped with a power utility-owned (commercial) smart meter and is linked via AMI to a smart grid, a HEMS has the ability to interact with individual electrical appliances via IoT devices such as power meters deployed and installed intrusively for them for the purpose of load management. Such a system allows power utilities to come up with DR policies for residential DSM. By doing so, the house environment conducting a HEMS does not run on guesswork for its load management. DSM comprises a collection of approaches which are used to improve energy efficiency, such as “time of use” and DR for demand-side regulation [19]. DR is an opportunity for customers to play a crucial role in reducing or shifting their electricity use in response to time-based tariffs or other forms of financial incentives [20]. Considering AMI in a smart grid, smart metering technology enables the prediction of future electricity demands, which can be used by policymakers to make decisions regarding increasing electricity demands. The ongoing development and deployment of smart meters [21,22] has made DSM that deals with ever-increasing electricity demands feasible [23,24]. HEMSs that optimize residential energy consumption in response to DR signals for DSM play an important role in a smart grid.

**Figure 1:**
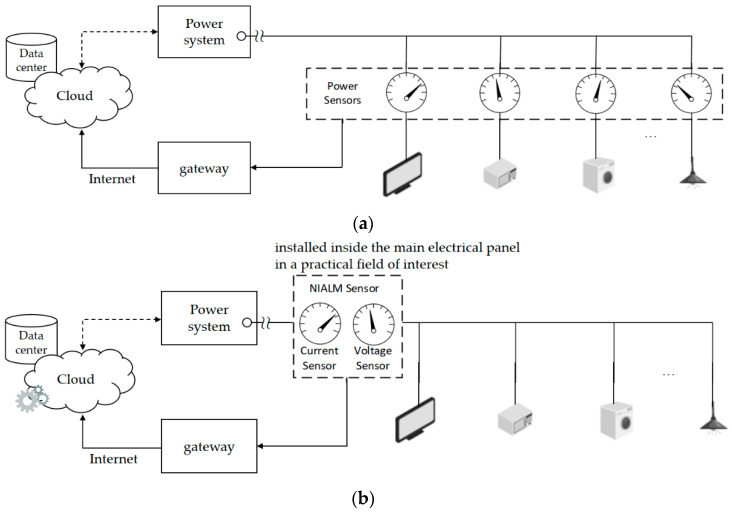
Appliance load monitoring approaches: (**a**) Intrusive appliance load monitoring (IALM); (**b**) non-intrusive appliance load monitoring (NIALM). Besides IALM, NIALM conducted in a practical field of interest such as a smart house can draw several inferences such as appliance-level energy consumption from total (circuit-level) energy consumption acquired by only one minimal set of plug-panel current and voltage sensors (or alternatively retrieved from a single source such as a power utility-owned smart meter [21,22]) instead of deployed and installed plug-load power meters (smart plugs) for relevant individual electrical appliances.

Figure 1 shows two different types of appliance load monitoring approaches, namely, intrusive appliance load monitoring (IALM, refer to Figure 1a) and non-intrusive appliance load monitoring (NIALM, refer to Figure 1b). The term “non-intrusive” points out the distinction to IALM. Although HEMSs can be used for residential DSM in a smart grid, households are instrumented (i.e., they are instrumental via deployment and installation of plug-load power meters). Power meters (smart plugs) must be deployed and installed intrusively for the relevant individual electrical appliances, as shown in Figure 1a. By contrast, NIALM, which is an efficient and cost-effective load monitoring approach developed for energy disaggregation in residential DSM contexts [25], makes it feasible for customers to keep track of fine-grained energy consumption of each relevant electrical appliance in their household without an intrusive deployment and installation of plug-load power meters. Based on individual characteristics of relevant electrical appliances, NIALM figures out a breakdown of whole-house energy consumption, where circuit-level energy consumption is decomposed into appliance-level (appliance-by-appliance) energy consumption by means of complex AI applied to composite (aggregate) current and voltage signals acquired by one single minimal set of plug-panel current and voltage sensors. NIALM can be part of an HEMS in a smart house in a smart grid, which has gained popularity in recent years for DSM (primarily in residential contexts). With NIALM, only one minimal set of current and voltage sensors installed at the electrical service entry point of a practical field of interest is required, as shown in Figure 1b. NIALM represents a minimum invasion of privacy [24] for energy disaggregation. NIALM has become more and more widespread in recent years [24].

Edge computing overhauls cloud computing—a centralized paradigm of AIoT [26,27]. The studies in [6,7,8,9,10,11,12,13,14,15,16,17,18] have featured HEMSs. However, the HEMSs that were implemented in [6,7,8,9,11,13,18] are based on cloud computing for residential DSM. Additionally, in [10], proposing a new framework of HEMSs based on both centralized energy disaggregation and distributed appliance controls, no practical evaluation is demonstrated for the evaluation of the feasibility of the framework as a whole. Moreover, the HEMSs that were implemented in [7,8,9] monitor relevant electrical appliances in an intrusive way. NIALM approaches that monitor relevant electrical appliances in a non-intrusive fashion have been developed significantly in [18,25,28,29,30,31,32,33,34]. These approaches can provide accurate energy disaggregation in many practical cases. Nevertheless, they leave NIALM developed over fog-cloud computing as part of an HEMS. The process of NIALM should be split into two stages: the first stage is that AI training is run on a powerful backend server serving as cloud computing for off-line training, and the second stage is that AI trained with a satisfactory level of performance in the cloud is run on an ARM^®^ (advanced reduced instruction set computing machine) processor-based embedded system, i.e., the core entity of a (residential) EMS, for the purpose of edge computing for on-line load monitoring. Edge computing overhauls cloud computing, extending the capabilities of cloud computing close to the edge of the network, and works as a collaborative learning technique with cloud computing for IoT applications. This technology has recently been developed and recognized as a promising, efficient decentralized paradigm of AIoT [26,35,36]. Edge computing, which aims to displace data science analytics in the cloud as close as possible to the edge of the network [27,37], can prevent network latency for latency-sensitive IoT applications (where network connectivity is not always available) [38,39] and fulfill the lack of location awareness as well as data mobility for IoT end devices [36] deployed in practical fields of interest (i.e., real-time responsiveness from heterogeneous sensor data for local interpretable and actionable data insights can be guaranteed). Edge computing has been foreseen as a remedy to alleviate the various issues of cloud computing [40,41]. The contributions of this study are summarized as follows.

**Figure 2:**
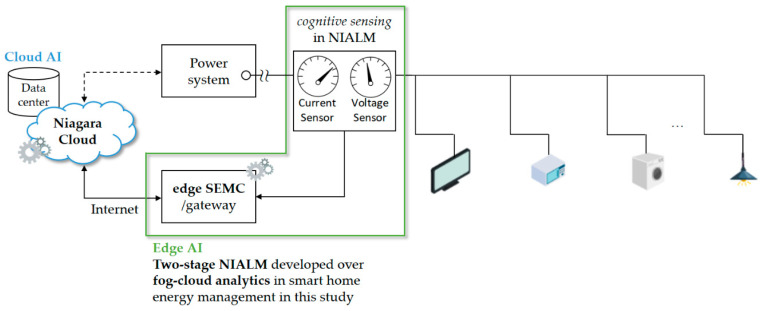
Developed NIALM approach over fog-cloud computing for smart home energy management in this study. Edge SEMC refers to the smart energy management controller considering edge computing.

- A newly developed smart home energy management system (SHEMS) prototype based on Tridium’s Niagara Framework^®^ has been developed and established over fog (edge)-cloud computing, which can scale to highly distributed systems made of tens of thousands of nodes/IoT end devices by embedded systems running the framework software for a large-scale implementation in energy management as an example.
- A two-stage NIALM has also been investigated in the framework, as shown in Figure 2. The newly developed SHEMS prototype utilizes the investigated two-stage NIALM to non-intrusively monitor relevant electrical appliances without an intrusive deployment of plug-load power meters (smart plugs). The complete NIALM approach comprises data acquisition, event detection and feature extraction, and load recognition, involving the following two stages:
  (1) An off-line training stage is achieved through AI (a deep-learning (DL)-accommodated artificial neural network (ANN)-based load recognizer in this study) that is trained and validated with a satisfactory level of performance in load recognition in cloud computing; and
  (2) An on-line load monitoring stage is processed on-site on the core entity of the SHEMS prototype, an ARM^®^ processor-based embedded system, as edge computing, where well-trained and -validated AI in the cloud is remotely deployed over the Internet at the edge of the network (real-time actionable insights can be made on-site).

In this study, the core entity of the SHEMS prototype, which operates as the operating nucleus of home energy management, is based on a compact, cognitive embedded IoT controller that connects IoT end devices such as sensors and meters. The controller serves as a gateway/SCADA (supervisory control and data acquisition) in a smart house/smart building for residential DSM. The core entity of the SHEMS prototype is named the “edge smart energy management controller” (eSEMC for short). As demonstrated and reported in this study, the established SHEMS prototype is feasible and usable, where the NIALM approach is realized completely over fog-cloud computing with Tridium’s Niagara Framework^®^ and LINE Notify for the third-party push notification service is integrated within the framework.

This study is structured as follows: the methodology of the SHEMS prototype having the complete two-stage NIALM is presented in Section 2; Section 3 shows the experimentation; finally, this study is concluded with some possible future research directions in Section 4.

## 2. Methodology

The developed SHEMS prototype considering a two-stage NIALM approach over fog-cloud computing is described in this section. Section 2.1 describes the SHEMS prototype based on Tridium’s Niagara Framework^®^. Section 2.2 describes the considered two-stage NIALM in the SHEMS prototype where it is established over fog-cloud computing.

### 2.1. The SHEMS Prototype Based on Tridium’s Niagara Framework^®^

**Figure 3:**
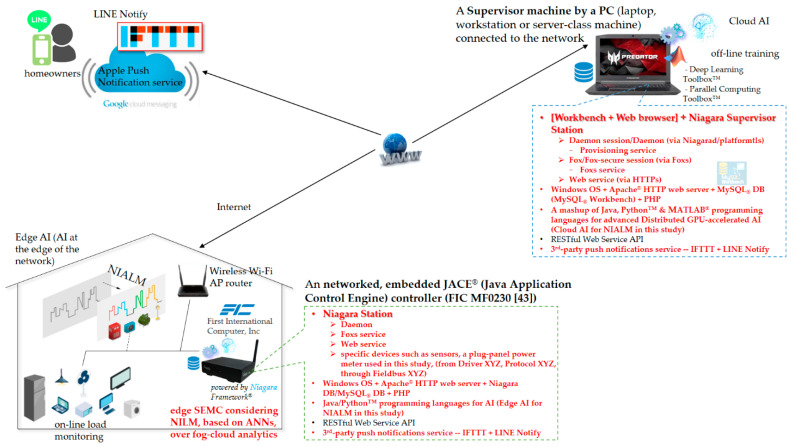
Developed smart home energy management system (SHEMS) prototype considering new two-stage NIALM over fog-cloud computing in this study, where the prototype is based on Tridium’s Niagara Framework^®^. The framework runtime is targeted for Java 8 SE compact3 profile compliant virtual machines (VMs). MATLAB^®^ is suited and used as the cloud here.

An HEMS in a smart house for residential DSM in a smart grid works as a bi-directional communication interface between demand and supply sides, where a power utility analyzes residential energy consumption data from grid-tied smart houses that are aware of DR signals and correspondingly optimize their power demands. A typical HEMS in a smart house for residential DSM in a smart grid is mainly made up of the following components: (1) a power company-owned (commercial) smart meter that communicates with a power utility’s cloud server via AMI. The meter is used to continually gather whole-house energy consumption data with a time resolution, for example, of 15 min (as expected, the NIALM methodology can be applied to smart meter data); (2) a central home gateway networked through the Internet with a 3rd-party cloud server where a broad range of energy services such as energy savings and energy conservation is provided for customers. The power utility’s cloud server features IP (internet protocol)-based DR programs which are published to and received by customers/the central home gateway for load management; and (3) the concerned/relevant electrical appliances that are monitored by individual plug-load power meters (smart plugs) and operated efficiently in response to DR programs for load management. As realized practically for residential DSM, an HEMS communicates with a cloud-centered server where the system has not been designed and implemented in consideration of fog-cloud computing. Additionally, the HEMS monitors electrical appliances in an intrusive manner, as plug-load power meters (smart plugs) are installed for individual relevant electrical appliances. Therefore, an SHEMS prototype considering NIALM over fog-cloud computing has been designed and implemented in this study. Figure 3 shows the system architecture of the designed and implemented SHEMS prototype based on the Niagara Framework^®^ considering a two-stage NIALM approach over fog-cloud computing. The prototype is based on Tridium’s Niagara Framework^®^. In the framework in Figure 3, an ARM^®^ processor-based embedded system, the core entity of the SHEMS prototype, is configured and used as edge computing. Furthermore, a cloud-centered machine, a laptop computer, is configured and used for cloud computing. The embedded system is networked via the Internet with the cloud-centered machine for the purpose of collaborative learning. Since the goal of residential DSM in a smart grid is to optimize customers’ power demands by electrical appliances, the SHEMS using the two-stage NIALM approach over fog-cloud computing identifies relevant electrical appliances in a non-intrusive fashion. The Niagara Framework^®^ [42] is a universal software infrastructure that allows companies/developers to build custom, web-enabled applications for managing, automating, and controlling smart IoT devices in real time over the Internet. Smart IoT devices include (1) monitoring and control devices, (2) sensors, (3) metering systems, and (4) embedded controls on packaged equipment systems. In this study, the Niagara Framework^®^ is conducted and further developed for energy service—NIALM for (residential) DSM, which is a part of building automation—automatic centralized control of a building’s HVAC (heating, ventilation and air conditioning), lighting, and many other devices. The Niagara Framework^®^, which uses the Java virtual machine (JVM) as its common runtime environment across various operating systems on different hardware platforms, is a Java software framework for integrating disparate building automation systems into a single, manageable interface that can be run on multiple hardware platforms [42]. The framework can scale from small embedded systems (embedded controllers developed as IoT end devices) to high-end servers (central machines configured as the cloud). Embedded systems that are configured, developed, and used in the framework are capable of running JVM. This excludes low-end devices without a 32-bit processor and several megabytes of RAM (random access memory). The framework can also scale to highly distributed (geographically distributed) systems made of tens of thousands of nodes (IoT end devices) running the framework software.

**Figure 4:**
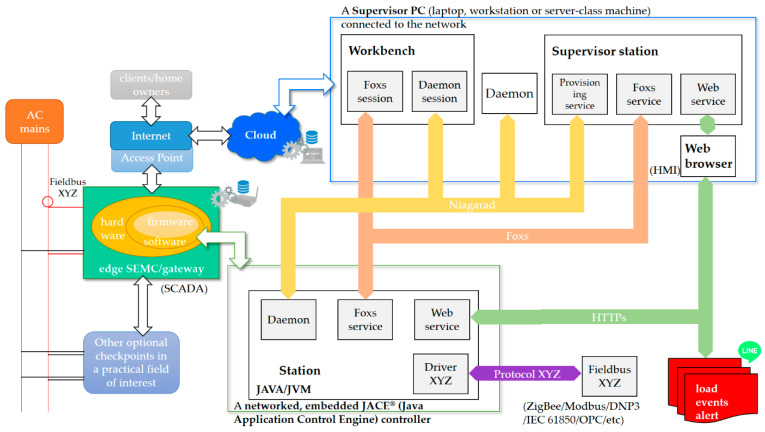
Configuration of the Niagara Framework^®^ that considers a supervisor PC/laptop and a single-networked, embedded JACE^®^ controller for SHEMS in this study.

The Niagara Framework^®^ can be hosted on a wide range of platforms, from small embedded systems (embedded controllers) to high-end servers, as mentioned above. Figure 3 also shows the simplest configuration of a single networked, embedded JACE^®^ (Java Application Control Engine) controller [42,43] that is integrated with a cloud-centered machine for NIALM over fog-cloud computing in this study, where the single-networked, embedded JACE^®^ controller is based on the FIC MF0230 controller [43] developed by First International Computer, Inc. [43]. Figure 4 shows the system configuration of the designed and implemented SHEMS prototype based on the framework consisting of a supervisor PC/laptop and a single-networked, embedded JACE^®^ controller in Figure 3, where multiple networked, embedded JACE^®^ controllers can be combined and managed in a distributed network for the purpose of monitoring multiple house environments, which includes a supervisor PC (laptop, workstation or server-class machine) that operates as a cloud-centered server for managing multiple-networked, embedded JACE^®^ controllers as edge computing and for providing potential for various user-centric IoT applications. In Figure 4, the networked, embedded JACE^®^ controller that collaborates together with the supervisor PC/laptop working as the cloud in the framework acts as a gateway (SCADA) named “eSEMC” for NIALM in this study for (residential) DSM.

A gateway, SCADA or data concentrator allowing for remotely monitoring and controlling relevant apparatus and machines involved in an automated process such as building automation collects data to be converted among different protocols such as Modbus, BACnet (building automation and control network), DNP3 (distributed network protocol 3), IEC 61850, and other legacy industrial communication protocols for the achievement of interoperability in (industrial) IoT, and is further analyzed in the cloud. Developed and conducted as edge computing, it can process collected data on-site for real-time actionable data insights provided for the purpose of automating the involved apparatus and machines intelligently. In this study, eSEMC working as edge computing in the framework is based on FIC’s MF0230 controller [43]. In this study, a two-stage NIALM approach has been developed in the framework over fog-cloud computing, where the immersion of AI into eSEMC, an ARM^®^ processor-based embedded system, for the purpose of disaggregating aggregated energy consumption data (or smart meter data) is achieved. eSEMC is referenced as a cognitive meter.

Figure 4 also shows the Niagara software processes and communication protocols for the networked, embedded JACE^®^ controller (i.e., eSEMC), which is remotely connected via the Internet with a supervisor PC/laptop running Workbench and managing the networked, embedded JACE^®^ controller in the framework. In the framework, the supervisor PC/laptop connected to the network is called a supervisor that acts as a cloud-centered server for managing the networked, embedded JACE^®^ controller (multiple embedded JACE^®^ controllers can be networked, combined, and managed in a distributed network for a large-scale demonstration of neighborhood-scale NIALM). In the framework, a wireless Wi-Fi AP (access point) router is configured.

As shown in Figure 4, the four different Niagara software processes associated with the framework are (supervisor) station, Workbench, Daemon, and Web browser [42]. They are detailed below.

- Station

A station is an instance of the framework running on a platform—a supervisor or an embedded JACE^®^ controller(s). It runs the Niagara components [42], pieces of self-describing software that can be assembled like building blocks to create new applications, of the (component-centric) framework. A station provides access for client browsers to view and control the Niagara components.

- Workbench

Workbench is an engineering tool, a JVM, that manages the Niagara components. A supervisor machine, which runs Workbench to accomplishes platform tasks such as launching and monitoring stations, has the tools to (1) serve as a central server (a single IP address) for delivering graphics and aggregated data for enterprise/user-centric IoT applications; (2) provide central database storage, a relational database management system (RDBMS) such as MS SQL, Oracle, MySQL^®^ or Hsql, for the purpose of collecting data from networked, embedded JACE^®^ controller(s); (3) act as an archive destination/repository for log and alarm data; and (4) provision (install/update) software modules including AI modules developed for an IoT application(s) in the cloud and deployed on-site on the networked, embedded JACE^®^ controller(s) as edge computing. Based on this framework, for NIALM, this study develops AI that is trained in the cloud for off-line learning and is deployed via the Internet on the networked, embedded JACE^®^ controller to support edge computing for on-line load monitoring. Niagara Cloud BaaS (backup as a service) [42] provides easy, secure, and scalable backups of Niagara stations on networked, embedded JACE^®^ controllers (as edge IoT end devices) to Niagara Cloud. This service can be used to remotely backup networked, embedded JACE^®^ controllers to a supervisor machine. Using Workbench on a supervisor machine as Niagara Cloud to networked, embedded JACE^®^ controllers, one can configure automated backups by time or event triggers, or initiate a backup manually. For history databases, networked, embedded JACE^®^ controllers as edge IoT end devices work with Niagara stations to collect data records at a regular specified time interval. The local storage capacity for histories by Niagara stations installed on networked, embedded JACE^®^ controllers is also specified for data records that are collected for and archived (exported/transferred) to Niagara Cloud. In this study, stores of NIALM data in a history database configured on the networked, embedded JACE^®^ controller at the edge of the network are committed regularly to Niagara Cloud where MySQL^®^ is suited.

- Daemon

Daemon is a native process, which is used to boot stations and manage platforms (for instance, platform configuration including IP settings is specified). Daemon functionality is accessed through Workbench.

- Web browser

A standard web browser such as Internet Explorer (IE) or Chrome hosts one of Niagara’s web user interfaces (UI) [42]. Niagara provides both server-side and client-side technologies to build a web UI/human machine interface (HMI).

In the configuration of the framework in Figure 4, three network protocols integrate the four different Niagara software processes. The three network protocols are Fox/Foxs (Fox-secure) [42], Niagarad/platformtls (secure Niagarad) [42], and HTTP (HyperText Transfer Protocol)/HTTPs (HyperText Transfer Protocol secure). These protocols are detailed as follows.

- Fox/Foxs

Fox/Foxs is a proprietary TCP/IP (transmission control protocol/Internet protocol) that is used for station-to-station and Workbench-to-station (i.e., the supervisor PC to the controller) communication.

- Niagarad/platformtls

Niagarad/platformtls is a proprietary protocol, which is used for Workbench-to-daemon communication. Additionally, in a supervisor station, it communicates with the provisioning service, which automates the execution of software modules developed for certain tasks and deployed on-site on remote-networked, embedded JACE^®^ controllers.

- HTTP/HTTPs

HTTP/HTTPs is a standard protocol used by web browsers to access station web pages. A web server can be configured on a supervisor PC, where RESTful (REpresentational State Transfer) web service can be provided and where the protocol is based on HTTP. Communications at the server side, a cloud-centered platform such as Microsoft Azure for data storage and data science analytics, can rely on a variety of protocols including HTTP, OPC (open platform communications), and SOAP (simple object access protocol)/MQTT (message queueing telemetry transport)/REST). The Niagara MQTT driver can be used to publish and subscribe Niagara points to Azure IoT hub, with the integration of Azure IoT Hub with the Niagara MQTT client.

One or more additional protocols (drivers) communicate with field-specific devices such as power meters (Modbus)/smart inverters (DNP3) for renewables (Driver XYZ, Protocol XYZ, and Fieldbus XYZ). In this study, a single-phase power meter (ICP DAS’s PM-3114 (Wiring: 1P4W-4CT) [44]), an IoT end device, is wired to the networked, embedded JACE^®^ controller (an IoT device), configured, and used to acquire aggregated RMS (root mean squared) power measurements, where acquired RMS power measurement data are transmitted from the power meter to the networked, embedded JACE^®^ controller (eSEMC) via the serial Modbus RTU (remote terminal unit) protocol over RS-485 and are then analyzed through the two-stage NIALM in the framework/SHEMS prototype for (residential) DSM. Developed in this study, the networked, embedded JACE^®^ controller with an immersion of AI (ANN-based two-stage NIALM) as edge computing is used to collect data (acquired aggregate RMS power measurements) and analyze collected data on-site for (residential) DSM, and it converts collected data from the different protocols if collected data to be further analyzed in the cloud are uploaded. In the framework, eSEMC, the core entity of the SHEMS prototype in Figure 3 and Figure 4, can be an ARM^®^ processor-based embedded system with a GPU (graphics processing unit) for the purpose of conducting DL. For example, it can be an nVIDIA^®^ Jetson Nano™ embedded system. In this sense, DL can be performed in consideration of edge computing to produce advanced AI for use in neighborhood-level NIALM. In the cloud, Deep Learning Toolbox™ and Parallel Computing Toolbox™ can be suited. Deep Learning Toolbox™ can provide a framework for designing and implementing deep neural networks that can be accelerated on a single- or multiple-GPU machine (with Parallel Computing Toolbox™) or even scaled up to clusters. Parallel Computing Toolbox™ can let AI developers solve computationally intensive problems based on multi-core computers, computer clusters, and/or GPUs.

Fog-cloud computing serves as converged computing for the purpose of knowledge sharing that consolidates IoT data from distributed IoT data aggregation [45]. For user-centric IoT applications developed in consideration of fog-cloud computing in smart houses/buildings, smart factories/manufacturers, and smart cities considering DSM in a smart grid, as expected, the developed SHEMS prototype will be able to perform converged computing for the demonstration of knowledge sharing where the prototype will be distributed over fog-cloud computing and be used to consolidate IoT data gathered from multiple fields of interest in a smart grid. Based on Tridium’s Niagara Framework^®^, the SHEMS prototype considering two-stage NIALM over fog-cloud computing in this study is a preliminary design and implementation towards such a scenario as a next-generation AMI. The two-stage NIALM over fog-cloud computing is presented in detail in the following subsection.

### 2.2. Two-Stage NIALM over Fog-Cloud Computing

**Figure 5:**
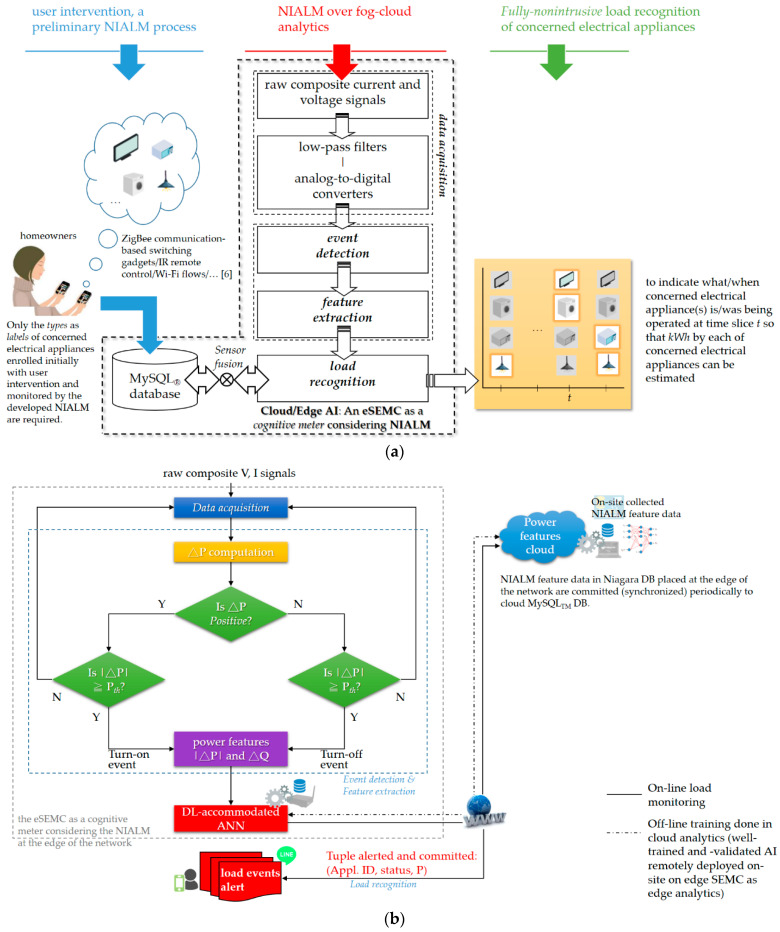
Investigation of the presented NIALM approach: (**a**) Pipeline of the two-stage NIALM, in simple terms, not requiring a one-time intrusive period for the preliminary NIALM process [6]; (**b**) the two-stage NIALM, based on the Niagara Framework^®^, over fog-cloud computing.

The presented NIALM approach is shown in Figure 5. Figure 5a depicts the two-stage NIALM in simple terms, considering an event-based NIALM approach over fog-cloud computing for collaborative learning, as illustrated in more detail in Figure 5b. The two-stage NIALM approach mainly performs data acquisition, event detection and feature extraction, and load recognition. Both composite electricity signals are simultaneously and continuously acquired through data acquisition, where they are measured by a single minimal set of current and voltage sensors, conditioned, and then digitized. Then, the acquired data are analyzed through event detection and feature extraction for load recognition. In the two-stage NIALM approach presented here, event detection is used to detect appliance events by abrupt power changes, gather NIALM feature data through feature extraction, and then label-gathered feature data for load recognition. For load recognition, AI that operates via a DL approach based on a feed-forward, multi-layer ANN trained through backpropagation here is used as a load recognizer in the two-stage NIALM, where the AI-based load recognizer decomposes the acquired total energy consumption data based on learned NIALM feature data. ANNs, connectionist systems, are computing systems that are inspired by biological NNs. The used DL approach based on a feed-forward, multi-layer ANN learns from on-site collected training data in the cloud. Load recognition is performed online once the AI model has been well-trained and -validated offline (i.e., it has been validated by test data with a satisfactory level of performance in load recognition). When an appliance event where |ΔP| (P refers to real power) is greater than or equal to a pre-determined threshold, Pth, is detected at time slice t, the well-trained AI model takes a feature reading and then recognizes it for relevant electrical appliances whose status is currently “on” or “off” for the purpose of on-line load recognition.

**Figure 6:**
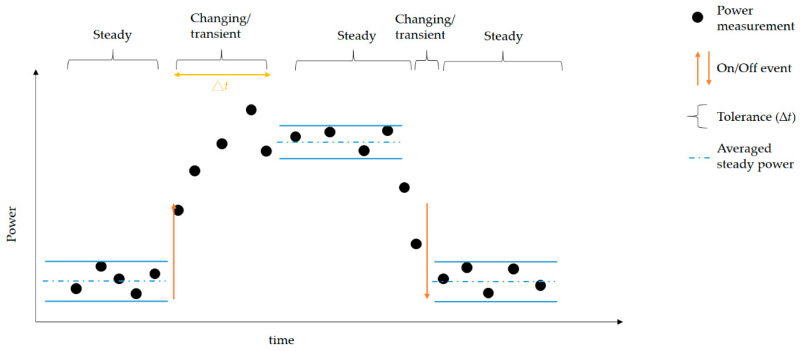
Detecting an appliance event in acquired total energy consumption through transient-passing event detection.

In the presented two-stage NIALM approach, features extracted from acquired composite current and voltage waveforms through feature extraction are |ΔP| and ΔQ (Q refers to reactive power), where ∆t is a slack parameter (which is chronologically scalable) for transient-passing event detection illustrated in Figure 6. The feature extraction process is based on the steady-state behavior of relevant electrical appliances.

In this study, the SHEMS prototype considering the two-stage NIALM utilizes a DL approach based on a feed-forward, multi-layer ANN that learns and distinguishes from extracted composite NIALM feature data for energy disaggregation, where the connectionism is trained through backpropagation in cloud computing for off-line learning and the well-trained and -validated connectionism is deployed remotely and executed on eSEMC as edge computing for on-line load monitoring.

**Figure 7:**
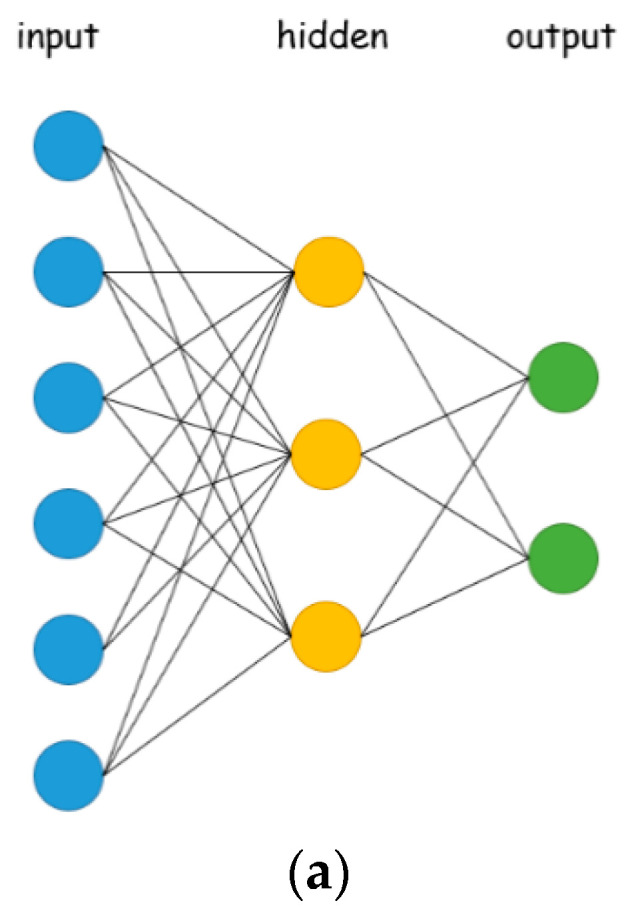

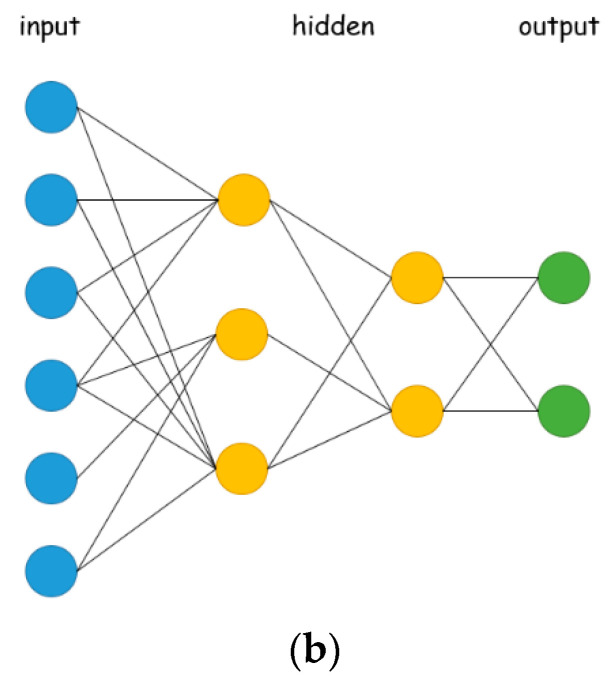
Representative artificial neural networks (ANNs): (**a**) An NN is fully connected; (**b**) An NN considers dropout—a hyperparameter whose value is specified and used to control the learning process of an NN—that can prevent overfitting [46] (another way used to prevent overfitting is early stopping).

ANNs have been developed from cognitive learning and information processing theories, seeking to imitate the learning behavior of the human brain and create complex interconnected neuronal structures. DL is usually based on an ANN architecture. The term “deep” refers to the number of (hidden) layers in the architecture; traditional ANNs contain only two or three layers, while deep NNs can have several layers, even hundreds. Figure 7 shows the general structure of an ANN. The general structure of an ANN is shown to those seeking to innovate in the application of ANNs in load management. As shown in Figure 7, a feed-forward, multi-layer ANN as a connectionism mimicking a biologic NN is made of an input layer, a number of hidden layers, and an output layer. The size of the input layer is dependent on the number of independent variables (i.e., extracted features) of considered feature data to be fitted. The number of hidden layers and the number of hidden neurons in each hidden layer can be experimented through hyperparameter tuning, where hyperparameters, comprising the learning mechanism and the number of epochs performed for iterative rounds of learning, affect how well the NN can represent its considered feature data. The size of the output layer is, usually, dependent on the number of dependent variables of considered feature data.

In this study, the F-measure, which is described in detail in the following subsection, is conducted and used as the performance metric to indicate the performance of the AI-based load recognizer in terms of load recognition.

### 2.3. Performance Evaluation by F-Measure for Load Recognition

As shown in Equation (1), the F-measure [47] is used to evaluate the performance, in terms of load recognition, of the developed SHEMS prototype having the presented two-stage NIALM.

(1) $$F-\mathrm{measure}=2\frac{\mathrm{Precision}\;\times \;\mathrm{Recall}}{\mathrm{Precision}\;+\;\mathrm{Recall}}$$

In Equation (1), precision stands for a ratio of the total number of correctly predicted positives to the total number of predicted positives. Recall (also known as the sensitivity or hit rate) accounts for a ratio of the total number of correctly predicted positives to the total number of actual positives. More details about precision and recall can be found in [47]. In summary, the F-measure is the harmonic mean of precision and recall, which is commonly used to evaluate a recognizer learning from a class-imbalanced dataset. A recognizer producing no false positives reaches a perfect precision. It also reaches a perfect recall when no false negatives are produced. Thus, a F-measure shown in Equation (1) and used to evaluate a recognizer can reach the best value, with perfect precision and recall, at 1.0. Besides the F-measure, we conduct receiver operating characteristic (ROC) curves [47] to evaluate the performance, in terms of load recognition, of the developed SHEMS prototype with the presented two-stage NIALM. A ROC curve consisting of the false-positive rate (FPR) and true-positive rate (TPR) produces a graph showing the performance, in terms of recognition, of a recognizer where it is examined at all recognition thresholds or with its different configuration settings [47]. In an ROC curve, an error to a trained and validated recognizer can be computed through the Euclidean distance, from obtained (FPR, TPR) to the perfect recognition (FPR = 0, TPR = 1) [29,30]. Equations (2) and (3) define FPR and TPR, respectively. TPR is referred to as recall.

(2) $$\mathrm{FPR}=\frac{\mathrm{FP}}{\mathrm{FP}\;+\;\mathrm{TN}},$$

(3) $$\mathrm{TPR}=\frac{\mathrm{TP}}{\mathrm{TP}\;+\;\mathrm{FN}},$$

In Equations (2) and (3), TP (true positives): the data observations are recognized, and they are associated to reality. TN (true negatives): the data observations are recognized, and they are associated to non-existence with the real. FP (false positives): the data observations are recognized as positives, but they are erroneously recognized against reality. Lastly, FN (false negatives): the data observations are recognized as negatives, but they are incorrectly recognized to existence in reality.

## 3. Experimentation

**Figure 8:**
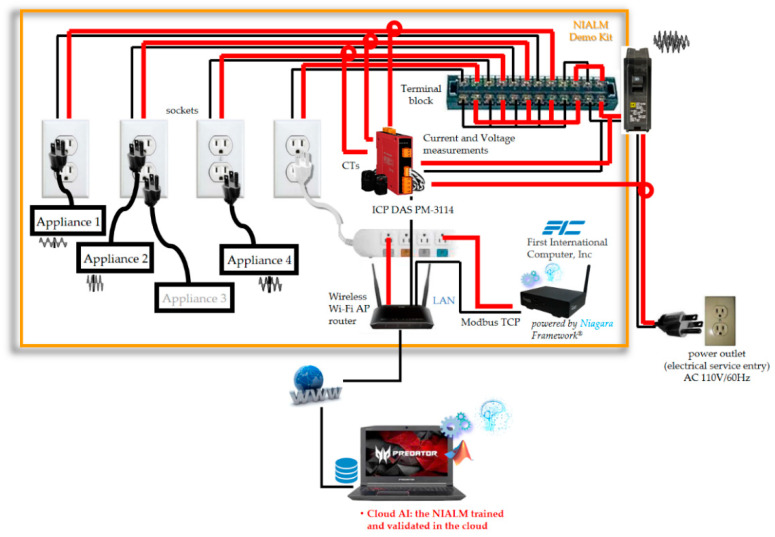

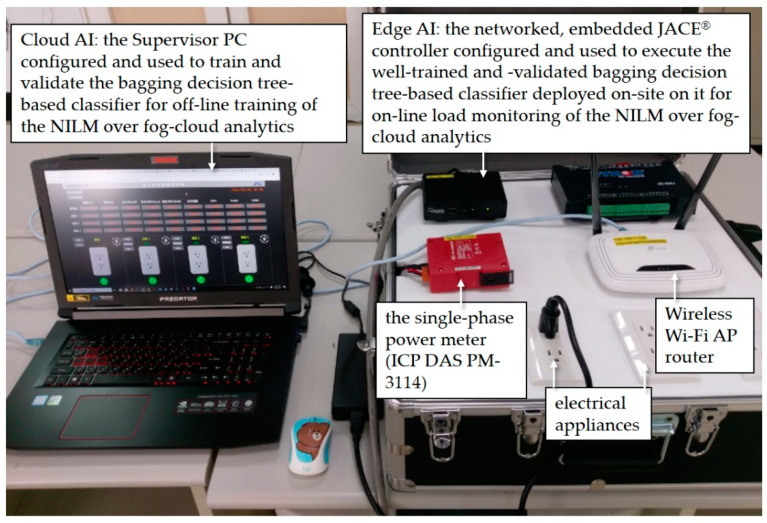
Experimental setup of the developed SHEMS prototype utilizing the presented two-stage NIALM approach over fog-cloud computing for the purpose of residential DSM. An electrical network topology with four power line branches in parallel is also shown.

In this section, the developed SHEMS prototype utilizing the presented two-stage NIALM approach over fog-cloud computing is demonstrated. Figure 8 shows its experimental setup, where it was experimentally evaluated in a laboratory environment. In this experiment, electrical appliances that were targeted, learned, and recognized through AI include a laptop, hair dryer, steamer, electric fan, and vacuum cleaner.

As presented in Section 2.2, the AI model used in the presented two-stage NIALM over fog-cloud computing is a DL approach based on a feed-forward, multi-layer ANN. As shown in Figure 8, a supervisor PC was configured as cloud AI and used to train and test the presented two-stage NIALM over fog-cloud computing for off-line learning of the targeted electrical appliances. A networked, embedded JACE^®^ controller (i.e., eSEMC) was configured as edge AI and used to execute a well-trained and -tested feed-forward, multi-layer ANN developed in the cloud and deployed on-site on the controller for on-line load monitoring of the targeted electrical appliances. A single-phase power meter (ICP DAS’s PM-3114 (Wiring: 1P4W-4CT) [44]) was installed and used for the purpose of acquiring aggregated RMS power measurements, where the acquired RMS power measurement data were transmitted from the power meter to the networked, embedded eSEMC (JACE^®^ controller/FIC’s MF0230) via the serial Modbus RTU protocol over RS-485. The received data were then recognized by a feed-forward, multi-layer ANN for on-line load monitoring, which was trained and validated with a satisfactory level of performance in terms of load recognition in the cloud for off-line learning.

In the design and implementation of the feed-forward, multi-layer ANN used as the load recognizer of the two-stage NIALM over fog-cloud computing here, the feedforwardnet() function in Deep Learning Toolbox™ [48] in MATLAB^®^ was used to create a two-layered feed-forward (shallow) ANN where (1) the connectionism was specified by one hidden layer with nine hidden artificial neurons, which was determined through trial and error. (2) The learning algorithm was based on the error backpropagation algorithm. (3) The cost function was the mean squared error (MSE) function. (4) The learning rate specified was 0.01, and (5) the specified maximum number of training epochs was 1000. The train() function was used to train the specified two-layered feed-forward ANN applied on training and test data. This function, train(), trains a shallow neural network, where an early stopping mechanism is provided and used to prevent overfitting. In this mechanism, the available dataset is divided into three subsets. The first subset is the training dataset, which is used to compute the gradient and update the network’s weight and bias coefficients. The second subset is the validation dataset. During the training process, the network trained for and applied on validation data is monitored. The error, MSE, obtained by the network monitored (i.e., applied on validation data) normally decreases in the initial phase of the training process. However, the error typically begins to rise when the network begins to overfit. When the error increases for a specified number of epochs, the training process is stopped. Additionally, the resulting weight and bias coefficients by the network trained and validated at the minimum of the error are returned. The third subset is the test dataset. During the training process, if the error obtained by the network validated for and applied on test data reaches a minimum, against the minimum of the error by the network applied on validation data, at a significantly different epoch, then this might indicate a poor division of the available dataset. For DL models [49], such as convolutional NNs, one should refer to the trainNetwork() function instead. ANNs, connectionisms as parallelisms, are inherently parallel algorithms. In Deep Learning Toolbox™ in MATLAB^®^, ANNs can be trained in parallel on CPU workers or GPU(s) with hundreds or even thousands of CPU cores involved in tens of thousands of concurrent threads. In this manner, parallel Computing Toolbox™ in MATLAB^®^ is required, and is used to allow ANNs to run across multiple CPU cores as workers on a single computer [50]. In this experiment, after the connectionism was well-trained and -tested in the cloud for off-line learning of the two-stage NIALM over fog-cloud computing, the connectionism was deployed on-site on the networked, embedded eSEMC working as edge computing for on-line load monitoring of the two-stage NIALM over fog-cloud computing. This process was used to obtain the on/off status of a targeted electrical appliance where its appliance event is detected and associated with its features reading to be taken, normalized, and recognized.

In this experiment, we divided a whole dataset into two disjoint parts of a training dataset and a test dataset. The training dataset, an available dataset for train(), was used to train the connectionism, while the test dataset was used to evaluate the trained, validated and tested connectionism in its performance in terms of load recognition. The obtained recognition rate was referred to as the outside-test recognition rate or the holdout recognition rate [51]. The so-called two-fold cross validation or the two-way outside-test recognition rate can be owned, where the whole dataset is divided into two disjointed parts of a training dataset and a test dataset with an equal size.

**Figure 9:**
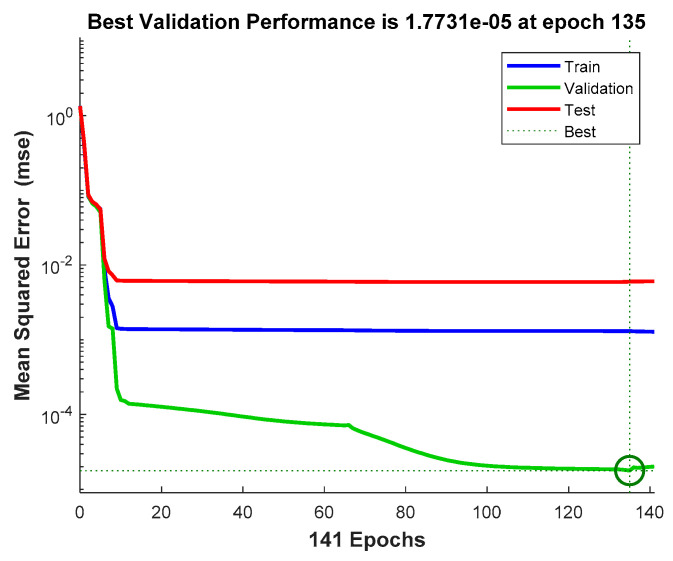
Training trajectory of the developed feed-forward, multi-layer ANN in this experiment, where the training process terminates at the 141-th epoch.

In this experiment, a total of 1206 data observations were collected on-site for off-line learning in the presented two-stage NIALM approach over fog-cloud computing for residential DSM, where the specified two-layered feed-forward ANN was trained, in the cloud, on 845 randomly sampled training data observations (~70% of the whole dataset). The trained ANN was then tested on the remaining 361 test data observations. In this experiment, a threshold, Pth, of 20.0 W was specified for transient-passing event detection, which is described in Section 2.2. Additionally, ∆t was specified as 7 s for feature extraction. Figure 9 shows the training trajectory of the specified two-layered feed-forward ANN, where the achieved MSE was 0.0013 with an initial MSE of 1.35. The specified two-layered feed-forward ANN can be trained across all available CPU workers on the supervisor PC. In the presented two-stage NIALM approach over fog-cloud computing, the power-intensive electrical appliances should not be operated at the same time, so the conductor of the electrical wiring is not overloaded.

Table 1 and Table 2 tabulate the load recognition results obtained by the trained feed-forward ANN applied on the training and test datasets, respectively. Table 3 and Table 4 show TPR values vs. FPR values for ROC curves obtained by the well-trained and -tested feed-forward ANN applied on the class-imbalanced data set in this experiment.

**Figure 10:**
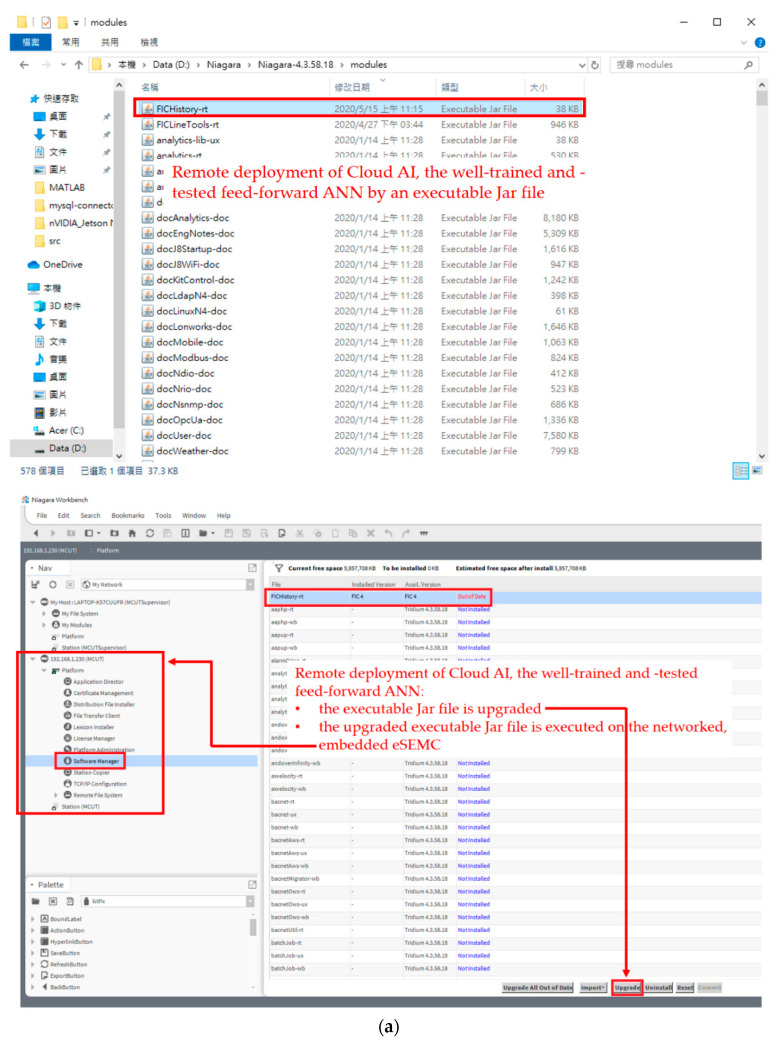

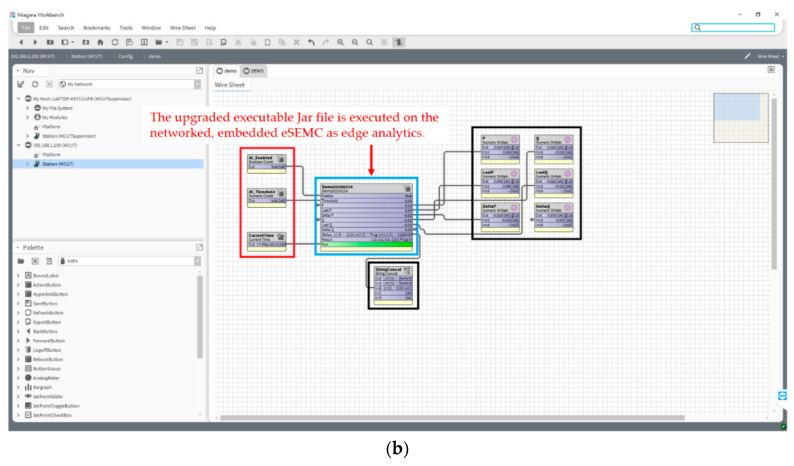
Remote deployment of the well-trained and -tested feed-forward ANN in cloud computing: (**a**) An executable Jar (Java ARchive) file was upgraded; (**b**) the upgraded executable Jar file is executed on the networked, embedded eSEMC as Edge AI.

Table 1 and Table 2 show that the data observations represent a class-imbalanced dataset. Accuracy alone does not show the full story by a recognizer trained on class-imbalanced data, where to each class there may exist a significant disparity between class positives (status on) and class negatives (status off). Therefore, the F-measure was conducted and used to fully evaluate the load recognizer, the trained feed-forward ANN, addressing the class-imbalanced dataset. Various metrics in addition to the F-measure have been developed. For AUCs in Table 3 and Table 4, one ROC curve can be shown per class. As reported in this section, the developed SHEMS prototype utilizing the presented two-stage NIALM over fog-cloud computing was able to distinguish from the targeted electrical appliances with an excellent level of performance in load recognition. As demonstrated here, the developed SHEMS prototype considering the presented two-stage NIALM over fog-cloud computing can discriminate relevant electrical appliances for (residential) DSM and is able to accommodate all types of ANNs including DNNs where their weight coefficients can be trained through distributed GPU computing in the cloud in the framework. The aim of this study is to develop an SHEMS prototype based on Tridium’s Niagara Framework^®^, where novel two-stage NIALM over fog-cloud computing is completed. The complete NIALM involves the following two stages. (1) An off-line training stage is achieved through AI (an ANN-based load recognizer in this study); AI is trained and validated with a satisfactory level of performance in load recognition in cloud computing. (2) An on-line load monitoring stage is processed on-site on the core entity of the SHEMS prototype (the networked, embedded eSEMC) as edge computing. Figure 10 shows the remote deployment of the well-trained and -tested feed-forward ANN in this experiment, where the well-trained and -tested feed-forward ANN in cloud computing was packaged as an executable Jar (Java ARchive) file, and was uploaded via Niagara Workbench to the remote networked, embedded eSEMC for on-line load monitoring in the presented two-stage NIALM over fog-cloud computing, as shown in Figure 10a. As shown in Figure 10b, the well-trained and -tested feed-forward ANN was deployed remotely on the networked, embedded eSEMC working as edge computing for on-line load monitoring in the presented two-stage NIALM over fog-cloud computing.

**Figure 11:**
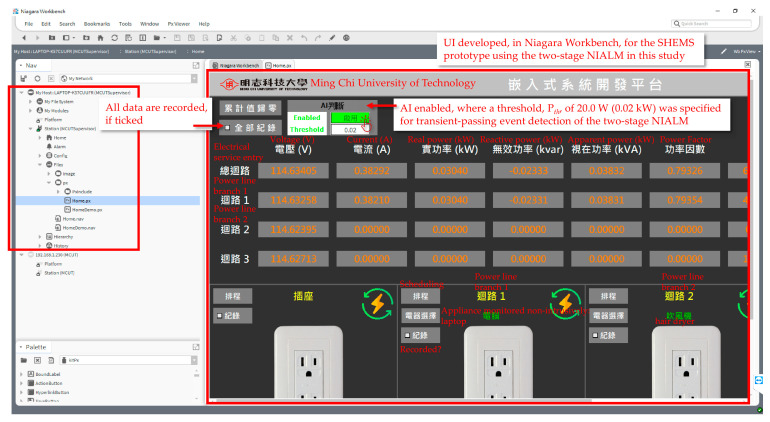
A user interface (UI) developed in Niagara Workbench for the established SHEMS prototype using the presented two-stage NIALM over fog-cloud computing in this study.

Figure 11 shows the UI developed in Niagara Workbench for the established SHEMS prototype having the presented two-stage NIALM over fog-cloud computing in this study, where (1) AI, the presented two-stage NIALM, is enabled with a threshold, Pth, of 20.0 W specified for transient-passing event detection of the two-stage NIALM; (2) all data can be recorded; and (3) power line branches can be recorded, monitored non-intrusively, and scheduled.

**Figure 12:**
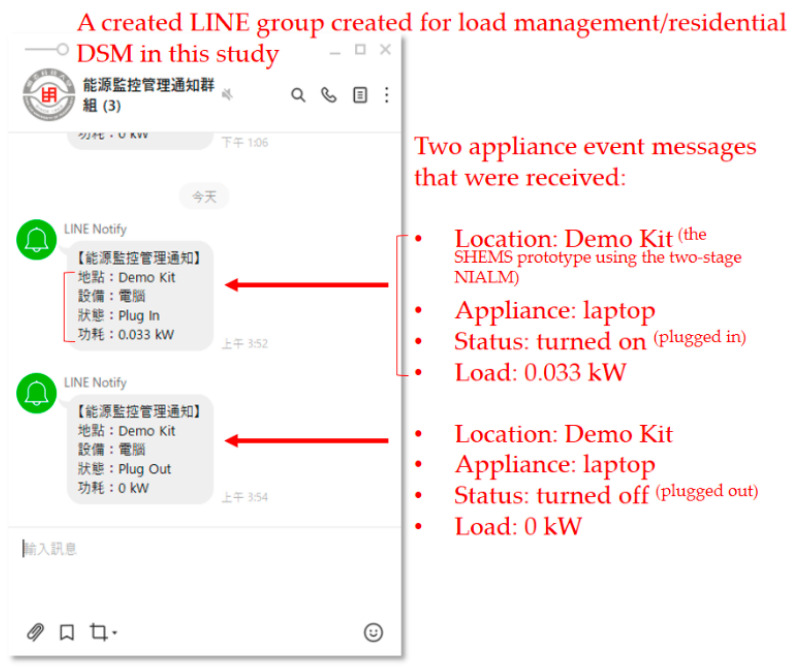
Appliance event messages received, in a created LINE group, by a LINE-Notify mobile phone over the Internet for load recognition via on-line load monitoring in the presented two-stage NIALM approach for load management/residential DSM in this study.

Regarding the push notification service in the developed SHEMS prototype with the presented two-stage NIALM over fog-cloud computing in this study, a practical paradigm of LINE Notify [52] was created, as shown in Figure 12 showing that appliance event messages were received, in a created LINE group, by a LINE-Notify mobile phone over the Internet for load recognition through on-line load monitoring in the presented two-stage NIALM approach in this study.

Members who are added in the LINE group can receive appliance event messages, by the SHEMS prototype using the two-stage NIALM, through integrated LINE Notify for load management/residential DSM in this study.

## 4. Discussion

The objective of this study is to develop an SHEMS prototype based on Tridium’s Niagara Framework^®^, where novel two-stage NIALM over fog-cloud computing has been completed. The prototype developed with the complete NIALM over fog-cloud computing is shown in Figure 8. The complete NIALM involves the following two stages:

(1) An off-line training stage is achieved through AI in the cloud. In this study, a DL approach based on a feed-forward, multi-layer ANN is considered for load recognition. As outlined in [46], providing a thorough investigation of DL/DNNs in its applications, mechanisms and uses in a variety of smart-world systems, DL/DNNs can improve investigation.
(2) An on-line load monitoring stage is processed on-site on the core entity of the prototype as edge computing. The core entity of the prototype is based on an ARM^®^ processor-based embedded system. Moreover, in the on-line load monitoring stage, a third-party push notification service by LINE Notify for receiving recognized appliance events in load management is integrated in the framework.

As experimentally evaluated in Section 3, the developed SHEMS prototype with the presented two-stage NIALM over fog-cloud computing was able to distinguish from the targeted electrical appliances with an average F-measure of 1.0 achieved in load recognition. The F-measure, which is the harmonic mean of precision and recall, was used to fully evaluate the performance of the prototype in load recognition. This is because the addressed NIALM is a class-imbalanced load classification problem. Besides the F-measure, AUCs representing the degree or measure of separability that the higher the AUC, the better the classification model is at predicting negative observations as class negatives and positive observations as class positives were also used. Additionally, Figure 10 shows the remote deployment of the well-trained and -tested classification model in the NIALM over fog-cloud computing, where the model was trained well in cloud computing, packaged as an executable Jar file, and uploaded via Niagara Workbench to the remote networked, embedded eSEMC working as edge computing for on-line load monitoring in the NIALM over fog-cloud computing. As shown in Figure 11, a UI developed in Niagara Workbench for the developed prototype with the NIALM over fog-cloud computing has been provided. It was provided for the design and implementation of the NIALM. Finally, for the third-party push notification service, LINE Notify for receiving appliance events recognized through on-line load monitoring in the NIALM for load management/residential DSM in this study has been integrated with the prototype in the framework, as shown in Figure 12.

## 5. Conclusions and Future Work

Electricity is an essential resource in today’s modern technologically driven society. In recent years, NIALM has become an alternative approach for efficient and cost-effective load monitoring for DSM in a smart grid. NIALM is an energy disaggregation technique that is used to analyze aggregated current and voltage measurements via a single minimal set of plug-panel current and voltage sensors for relevant electrical appliances for the purpose of load management. This study has developed an SHEMS prototype using novel two-stage NIALM over fog-cloud computing. The developed prototype is based on Tridium’s Niagara Framework^®^. In the two-stage NIALM presented in the prototype, a complete NIALM pipeline over fog-cloud computing has been investigated in consideration of data acquisition, event detection and feature extraction, and load recognition involving (1) off-line learning achieved by AI (a DL approach based on a feed-forward, multi-layer ANN) in cloud computing and (2) on-line load monitoring performed on-site on a networked, embedded eSEMC (which is the core entity of the SHEMS prototype with an immersion of well-trained AI) as edge computing and alerted through LINE Notify for third-party push notification service. The preliminary NIALM process shown in Figure 5a needs to be performed when a new electrical appliance is installed, where a re-training process for the trained ANN is involved with user intervention in the off-line learning stage of the two-stage NIALM. As demonstrated and reported in this study, the developed SHEMS prototype utilizing the presented two-stage NIALM over fog-cloud computing was able to distinguish from the targeted electrical appliances with an average F-measure of 1.0 achieved in load recognition. Additionally, the remote deployment of the well-trained and -tested model in cloud computing has been demonstrated, where the model was deployed remotely on the networked, embedded eSEMC working as edge computing for on-line load monitoring in the presented two-stage NIALM over fog-cloud computing. In addition, the LINE Notify-integrated third-party push notification service for receiving recognized appliance events in load management has been implemented in the framework. In summary, the developed SHEMS prototype with the presented two-stage NIALM over fog-cloud computing is feasible and usable. It can discriminate between electrical appliances in a non-intrusive way for (residential) DSM, where all types of ANNs including DNNs where their weight coefficients can be trained through distributed GPU computing in the cloud in the framework can be accommodated for further research. The choice of AI models for load recognition is not the main focus of this study. As outlined in [46], providing a thorough investigation of DL/DNNs in its applications, mechanisms and uses in a variety of smart-world systems, DL/DNNs can improve investigation.

The Niagara Framework^®^ conducted in this study can provide a scalability to a highly distributed system architecture, handling tens of thousands of IoT nodes (i.e., networked, embedded JACE^®^ controllers running the framework software) in practice. As expected in the future, the developed SHEMS prototype based on the Niagara Framework^®^ will be designed and implemented in a large-scale demonstration of the presented two-stage NIALM over fog-cloud computing to provide an intelligent power infrastructure, called iPower, for managing electricity consumption, where neighborhood-level NIALM gathering NIALM feature data from and leveraging gathered feature data across multiple practical fields of interest will be investigated in a big-data approach for (residential) DSM. Additionally, a stress test will be examined. As electrical appliances draw distorted, non-sinusoidal currents due to their inherent physical characteristics or the presence of power electronics, harmonics [28] in composite current signals can be extracted through fast Fourier transform (FFT) and used as additional electrical features, input variables of an AI model used as a load recognizer for NIALM, to distinguish electrical appliances that are identical in ΔP and ΔQ draws. With the use of FFT extracting harmonics/total harmonic distortion (THD) from acquired composite current signals, the sampling rate of data acquisition can be specified at 1 kHz or higher, which complies with the Nyquist–Shannon sampling theorem, where the Nyquist–Shannon sampling theorem shows that, to be able to sample a signal, the sampling frequency specified needs to be at least twice the frequency of the signal we are trying to sample. FFT applied on the sampled signal will only be capable of detecting frequencies up to half the sampling frequency. Harmonics/THD can be considered in the future as an additional set of steady-state electrical features for load recognition in the presented two-stage NIALM over fog-cloud computing (most electrical appliances, such as refrigerators [32], have distinguishable characteristic patterns. However, not all electrical appliances, such as kettles and washing machines, have distinguishable characteristic patterns [53], which makes load recognition in NIALM a difficult task). Finally, providing for domestic customers and further developed in the future, the presented two-stage NIALM over fog-cloud computing can provide services, such as the provision of services in the fault detection and diagnosis of rotating machinery, for industrial as well as commercial customers, where the prediction of the remaining useful life of relevant equipment monitored and modeled with the past trends from gathered historical data can be achieved.

## Figures and Tables

**Table 1:** Load recognition results obtained by the specified and trained feed-forward ANN in training.

	Precision	Recall	F-Measure	The Number of Training Data Observations
laptop	1.0	1.0	1.0	29
hair dryer	1.0	1.0	1.0	333
steamer	1.0	0.95	0.97	73
electric fan	0.99	1.0	0.99	397
vacuum cleaner	1.0	1.0	1.0	13
average/total	1.0	1.0	1.0	845

**Table 2:** Load recognition results obtained by the specified and trained feed-forward ANN in test.

	Precision	Recall	F-Measure	The Number of Test Data Observations
laptop	1.0	1.0	1.0	11
hair dryer	1.0	1.0	1.0	149
steamer	1.0	1.0	1.0	24
electric fan	1.0	1.0	1.0	172
vacuum cleaner	1.0	1.0	1.0	5
average/total	1.0	1.0	1.0	361

**Table 3:** Obtained TPR values vs. FPR values for ROC curves by the well-trained and -tested feed-forward ANN applied on the class-imbalanced training data in this experiment, where the AUC values are also shown for each class.

	FPR	TPR	AUC ^1^
laptop	0.0	1.0	1.0
hair dryer	0.0	1.0	1.0
steamer	0.0	0.95	0.97
electric fan	0.01	1.0	0.99
vacuum cleaner	0.0	1.0	1.0

^1^ The maximum AUC (area under the curve) is 1, which corresponds to a perfect recognizer—100% sensitivity of no false negatives and 100% specificity of no false positives.

**Table 4:** Obtained TPRs vs. FPRs for ROC curves, by the well-trained and -tested feed-forward ANN applied on the class-imbalanced test data in this experiment, where for the five load classes’ AUCs obtained are also shown.

	FPR	TPR	AUC
laptop	0.0	1.0	1.0
hair dryer	0.0	1.0	1.0
steamer	0.0	1.0	1.0
electric fan	0.0	1.0	1.0
vacuum cleaner	0.0	1.0	1.0

## Data Availability

Not applicable.

## Nomenclature

DSM	demand-side management
NIALM	non-intrusive appliance load monitoring
IoT	Internet of Things
SHEMS	smart home energy management system
AI	artificial intelligence
AIoT	AI across IoT
AMI	advanced metering infrastructure
DR	demand response
IALM	intrusive appliance load monitoring
ARM	Advanced Reduced instruction set computing Machine
ANN	artificial neural network
SCADA	supervisory control and data acquisition
eSEMC	edge smart energy management controller
JVM	Jave virtual machine
JACE	Java application control engine
RDBMS	relational database management system
REST	REpresentational State Transfer
DL	deep learning
FPR	false positive rate
TPR	true positive rate
ROC	receiver operating characteristic
